# A Case for a Diagnosis*Presented before the 8th Dist. Branch, N. Y. State Medical Society, Olean, N. Y. Sept. 22d, 1915

**Published:** 1915-11

**Authors:** J. Henry Dowd

**Affiliations:** Buffalo, N. Y.


### A Case For a Diagnosis.*

By J. HENRY DOWD, M. D., Buffalo, N. Y.

The following case I show you for two reasons: first, for a diagnosis, and secondly, to show what has been accomplished after practically every other method had failed during two years constant treatment, in which the patient could not work and suffered intense pain.

G. R. aet, 28, Bradford, Penna., married, gave the following history:

Ailing for two years: commenced by feet becoming sore on the bottoms and constant aching of the heels. Following this, he became very nervous: could not sit in room with his wife sewing or using scissors, thought she was going to stab him in the nose or face. Same sensation occurred if he saw a person using a knife (I used my knife to sharpen a pencil while he was in my office, as soon as he saw the knife, he shook as though having a chill). Said he was perfectly aware no injury would be done him, but when first saw sharp instruments, would unconsciously shake and become terror stricken, these impressions were gradually becoming worse; he was almost afraid to go out of doors. Following this his fingers and feet, (phalanges), became very painful and intensely sore. Gradually this same condition appeared in the cervical vertebra: at consultation he could only bend or turn his head with the greatest difficulty. There was an occasional pain in elbows, hip and knees, but these seemed only transitory. When he would reach for anything, as a book, his pipe and the like, about midway in his effort the extending arm would suddenly become arrested—locked so to speak. This was only for an instant and unaccompanied by pain, but would always occur. Veins of the legs would swell out like ropes, and apparently hard knots appear at certain spots: the patellas were greatly exaggerated, ankle clonus was absent.

He was only able to sleep about four hours out of thirty-six. The joints were all swollen to about twice their natural size, but by massage, he could reduce them to almost normal: the swelling at examination appeared to be involvent of the bone, but there was no lateral movement.

**Previous History.** Typhoid, five years before, in bed five or six weeks. Never venereal disease, smokes, does not drink, erections good. About two years previous to attack, underwent severe nervous strain for some time, worried constantly.

**Family History.** Mother and father alive, never had rheu-

*Presented before the 8th Dist. Branch, N. Y. State Medical Society, Olean, N. Y., Sept. 22d, 1915

matism: but mother a marked neurotic, father also of a nervous temperament, but no similar conditions in either.

**Urinary Examination.** One and three quarters ounces of urine passed in five hours: sp. g. 1028, trace of albumin, no pus, casts, sugar, blood, bacteria or crystals: indican slightly increased, phosphaltic index 90% plus, solid: crystals normal in character, but small: hemoglobin, 90%.

**Treatment.** Of course, this was imperical. For two years he had aspirin, the salicylates, electricity, phylacogens, even X-ray, all a dismal failure: He was gradually becoming worse. The index being 90% plus, nerve-cell irritation was evident, and the case being chronic, he was placed on the Bromide of gold and arsenic.

In three weeks the following had resulted: sleeping about eight hours every night, pain decidedly lessened, fear of sharp instruments almost absent, fingers do not swell as often, and then but little. Urinary examination at this time about the same excepting, sp. g. 1015, albumin less, phosphatic index, 40% minus: He was passing about two ounces every hour and a half. The index showed plainly excessive nerve metabolism had ceased and there remained only about 40% nerve nourishment as a residual: in other words, nerve cell impoverishment was now plainly evident. He was placed on Comp. Phos. Tonic (Dowd) Oz. 2, Fl. Ex. Valerian, Oz. 1, in glycerine qs, ad Oz. 6. One teaspoonful in milk about half an hour after meals. From this time on, there was steady improvement until now, although joints of fingers are swollen, due to bony involvement, there is scarcely any pain: entire freedom from pain in the cervical region, can move head freely. There is also entire absence of locking of elbows: sleep sound every night, good appetite. He has worked every day for the past five months, including Sundays. There is no fear of sharp instruments, says he feels 90% better than he was six months ago. Urinary examination shows sp. g. 1020, no albumin, no pus, no bacteria or sugar: index 10% plus.

No diagnosis of this case will be offered, Acromegaly, gigantisme, Tomsesn's disease and rheumatic arthritis can be eliminated. It may possibly be arthritis deformans, but many of the cardinal symptoms of this disease are absent. The writer offers only a tentative cause, irritation of the pitutiary body: there is no infective process evident.

40 North Pearl Street.

---

**Carcinoma of Stomach in Man Aged 26.** A hard, nodular growth, of the posterior wall and greater curvature, invading gastro-colic omentum and transverse colon, found to be inoperable, apparently of 2 years' duration. Wm. Young, N. Zealand Med. Jour., Aug.

